# Mechanical and physio-biological properties of peptide-coated stent for re-endothelialization

**DOI:** 10.1186/s40824-020-0182-x

**Published:** 2020-01-23

**Authors:** In-Ho Bae, Myung Ho Jeong, Dae Sung Park, Kyung Seob Lim, Jae Won Shim, Mun Ki Kim, Jun-Kyu Park

**Affiliations:** 1 Cardiovascular Convergence Research Center of Chonnam National University Hospital Designated by Korea Ministry of Health and Welfare, Jaebongro 671, Dong-gu, Gwangju, 61469 Republic of Korea; 2 Korea Cardiovascular Stent Research Institute, Jangsung, 57248 Republic of Korea; 3 Department of Cardiology, Chonnam National University Hospital, Gwangju, 61469 Republic of Korea; 4 Cardiovascular Convergence Research Center of Chonnam National University Hospital, Gwangju, 57248 Republic of Korea; 5 National Primate Research Center & Futuristic Animal Resource and Research Center, Korea Research Institute of Bioscience and Biotechnology, Ochang, 28116 Republic of Korea; 6 CGBio Co. Ltd., Jangseong, 57248 Republic of Korea

**Keywords:** Peptide-coated stent, Mechanical properties, Drug-eluting stent, Re-endothelialization, Peptide delivery

## Abstract

**Background:**

The aim of this study was to characterize the mechanical and physio-biological properties of peptide-coated stent (PCS) compared to commercialized drug-eluting stents (DESs).

**Methods:**

WKYMVm (Trp-Lys-Tyr-Met-Val-D-Met), a stimulating peptide for homing endothelial colony-forming cell was specially synthesized and coated to bare metal stent (BMS) by dopamine-derived coordinated bond. Biological effects of PCS were investigated by endothelial cell proliferation assay and pre-clinical animal study. And mechanical properties were examined by various experiment.

**Results:**

The peptide was well-coated to BMS and was maintained and delivered to 21 and 7 days in vitro and in vivo, respectively. Moreover, the proliferation of endothelial cell in PCS group was increased (approximately 36.4 ± 5.77%) in PCS group at 7 day of culture compare to BMS. Although, the radial force of PCS was moderated among study group. The flexibility of PCS was (0.49 ± 0.082 N) was greatest among study group. PCS did not show the outstanding performance in recoil and foreshortening test (3.1 ± 0.22% and 2.1 ± 0.06%, respectively), which was the reasonable result under the guide line of FDA (less than 7.0%). The nominal pressure (3.0 mm in a diameter) of PCS established by compliance analysis was 9 atm. The changing of PCS diameter by expansion was similar to other DESs, which is less than 10 atm of pressure for the nominal pressure.

**Conclusions:**

These results suggest that the PCS is not inferior to commercialized DES. In addition, since the PCS was fabricated as polymer–free process, secondary coating with polymer-based immunosuppressive drugs such as –limus derivatives may possible.

## Introduction

Coronary artery stent implantation is annually used to treat more than 1 million patients with obstructive coronary artery disease. However, the infrequent yet important problems of in-stent restenosis and stent thrombosis limit clinical efficacy. With the advent of drug-eluting stents (DESs) which are coated with anti-cancer or immunosuppressive drugs, neointima growth and revascularization rates are lower compared with bare metal stents (BMSs) [1]. These drugs, however, markedly inhibit endothelial cell proliferation and delay re-endothelialization, resulting in acute stent thrombosis [2]. As well as, recent clinical studies suggest that DESs delay re-endothelialization and, in some studies, appear to be accompanied by a higher prevalence of stent thrombosis [3]. Conversely, increased re-endothelialization after stent implantation correlates with reduced neointima formation. In other word, endothelial injury by stent implantation and incomplete re-endothelialization lead to the onset and progression of severe vascular disorders, such as neointimal hyperplasia, atherosclerosis, and acute/late stent thromboses of injured arteries [4, 5].

WKYMVm (Trp-Lys-Tyr-Met-Val-D-Met), a selective formyl peptide receptor (FPR)-2 agonist have been reported to be a potent stimulant of leukocyte [6], leucocytes seven-transmembrane receptor [7], and neovasculogenesis factor [8]. Especially, WKYMVm stimulated chemotactic migration, angiogenesis, and proliferation ability of endothelial colony-forming cells (ECFCs) [8]. Moreover, the G-protein-coupled FPR-2 has been implicated in regulation of inflammation and angiogenesis [9]. Based on these reports, it was postulated that the re-endothelialization after stent implantation may be enhanced by stimulating homing of ECFCs via peptide released from coronary stent.

Even though stents are routinely used in the majority of cardiovascular catheterization procedures to treat stenotic arteries, their clinical effectiveness is hindered by numerous post deployment complications such as biocorrosion and structural failure [10, 11], which may lead to inflammation, thrombosis and ultimately in-stent restenosis [12]. A large number of stents with different geometric and mechanical features are available on the market. The therapeutic efficacy of stents depends largely on their mechanical properties [13, 14]. Therefore, the mechanical properties of stents influence the choice of stents for treating specific lesions [15]. The mechanical characteristics of an ideal stent have been described in numerous reviews [16–18]. Such a stent should have the ability to be crimped on the balloon catheter, good expandability ratio, sufficient radial hoop strength and negligible recoil, sufficient flexibility, adequate radiopacity/magnetic resonance imaging (MRI) compatibility, thromboresistivity, and drug delivery capacity. This has become one of the indispensable requirements for contemporary stents in an effort to prevent restenosis [18]. The optimization of material properties, surface finish, and stent design is necessary for the development of stent [19]. It is interesting to note that many DES essentially employ the same design configuration as their BMS counterparts with a drug-loaded polymer coating on the underlying metallic surface. These similarities have led to some concern over the long-term effect of DES following complete elution of the drug [20]. That is the reason why, previously, we have reported that our own stent design (designated as CNUH stent) to prepare the backbone for the development of DES [21, 22]. It was greatly superior to other commercialized stent in terms of stent flexibility. Moreover, in animal study, the CNUH stent was not inferior to commercialized BMS on various histological parameters. In this study, the WKYMVm peptide was coated on CNUH stent (designated as PCS). Thereafter they were subjected to mechanical performance tests and compared to commercialized DESs. In addition, physio-biological properties such as peptide delivery and cytotoxicity were investigated.

## Materials and methods

### Investigated stents

Four commercialized stents {Xience Prime™ (Abbott), PROMUS Element™ (Boston Scientific), Endeavor Resolute Integrity™ (Medtronic), and Biomatrix™ (Biosensors)} and PCS were prepared and subjected to mechanical performance test. All experiments were performed with same diameter of stents (3.0 mm).

### Preparation of bare metal stent

Previously, we have reported that the own designed bare metal stent (BMS, designated as CNUH stent) [21, 22] . It showed the great performance on flexibility, biocompatibility and moderate performance under the guide line of FDA in foreshortening and recoil performance. There is no doubt that CNUH stent is enough competitive compared to the other competitor. Therefore, it was utilized as a platform for PCS. Briefly, the Cobalt-Chromium (Co-Cr, 3.0 mm × 18.0 mm, L605) alloy was used as a stent material because many researchers have reported that the Co-Cr is the most appropriate material in terms of biocompatible aspects [23]. Fabrication of the BMS with the Co-Cr alloy was performed using a laser cutter (Rofin, Starcut, Hamburg, Germany). Thereafter, it was applied in acidic atmosphere (50% H_2_SO_4_) for 1 h to remove and crush of burr. And then heat treatment and polishing process was performed to restore the mechanical properties. The cleaned BMS was kept under vacuum oven at 60 °C for 2 h to evaporate the residual water.

### Fabrication of peptide-coated stent

The WKYMVm was synthesized and purified by Anygen (Gwangju, Republic of Korea), according to the published sequence [24]. The fluorescence marker (FITC) was conjugated to lysine moiety. The purity of synthesized peptide was greater than 90%, and the amino acid composition was verified by mass spectrometer. To immobilize the peptide to BMS surface, the dopamine which is the composition of adhesive chemical in mussels was employed. As we have reported previously [25, 26], we synthesized peptide dopamine conjugates using NHS and EDC as an activation agent of carboxyl group of peptide. Briefly, peptide (2 mg), NHS (50 mM, 1.15 mg), and EDC (100 mM, 3.83 mg) were dissolved in MES buffer (50 mM, pH 5.5, 2 ml) to minimize hydrolysis of EDC [27]. The solution was stirred at room temperature for 2 h, and dopamine (15 mM, 0.6 mg) was added to the solution. The resulting mixture was stirred at room temperature for an additional 8 h. The resultant solution was transferred to molecular porous dialysis membrane bags and underwent dialysis for 12 h to remove unreacted coupling reagents. To prevent dopamine from self-polymerization, 5 mM of HCl was added to the dialysis water. The solution was freeze-dried to obtain the solid-state compound. The compound was completely dissolved in tris buffer (10 mM, pH 8.5). BMS were immersed in the peptide solution. After 10 h incubation, the specimens were taken out and sufficiently washed with water.

### In vitro release of peptide

In vitro release kinetics was investigated by optical analysis of FITC which was conjugated to peptide. Unlike many studies, which used a simple shaking procedure [28], the appropriate equipment was designed for this study, mimicking the body’s circulation system (data not shown). A peristaltic pump (JenieWell, Seoul, Korea) and various thicknesses of silicon tubing were used to function as the heart and vasculature of the body, respectively. Three stents of PCS were inserted and then expanded in the silicon tubing using a balloon (3.0 × 20.0 mm) with under 9 atm of pressure. And the phosphate buffered saline was circulated through the tubing by dipping both open ends of the silicon tubing in the temperature controlled reservoir. The circulating rpm was set at 150, and a one-directional flow was used to simulate the body’s circulation system. At every designated time point, the PCSs were taken out and subjected to fluorescence microscope observation.

### Morphometric characteristics

The surface images of PCS and peptide released to vessel surrounding stents were analyzed fluorescence microscope (BD biosciences, Franklin Lakes, NJ. USA). The surface morphologies of stents were evaluated by scanning electron microscope (SEM; HITACHI, Tokyo, Japan). “Prior to cell imaging, the samples were fixed and dehydrated. The Thio-CS was soaked in the primary fixative of 2.5% glutaraldehyde (Sigma) for 2 h. And then the samples were then dehydrated by replacing the buffer with increasing concentrations of ethanol (from 40 to 100%) for 10 min each. Consequently, the samples were dried at room temperature for 24 h. All samples were subjected to SEM at voltages ranging from 5 to 15 kV after the samples were sputter-coated in white gold.”

### In vitro endothelial cell proliferation

To investigate the effect of peptide-coated stent on endothelial cell proliferation, human umbilical vein endothelial cell (HUVEC; Lonza, Rockland, ME) was employed. The stent were immersed in cell culture media as a 2-dimentional shape. Thereafter, HUVECs were seeded at 1 × 10^4^ cells/cm^2^ in a 24-well culture dish. The culture dishes were incubated at 37 °C in a humidified 5% CO2 atmosphere. The proliferation of cells was evaluated by XTT assay using an EZ-cytox cell viability assay kit (Daeil Lab Service Co., Seoul, Korea). Briefly, 40 *ul* of EZ-cytox reagent was added to a 24-well culture dish. By the action of mitochondrial dehydrogenases, XTT is metabolized to form a formazan dye that can be spectrophotometrically determined by measuring the absorbance at 450 nm using a spectrophotometric microplate reader (Bio-Tek Instruments, Winooski, VT). The amount of formazan salt formed corresponds to the number of viable cells contained in each well [29] .

### Mechanical performance study

The flat plate compression and 3-point bending test which are providing radial force and flexibility respectively were carried out. Furthermore, it undertakes foreshortening and recoil test with simple mathematic equations by obtaining the values of length and radius in the stent before and after expansion. Nominal pressure of stents accessed by compliance performance provides a reasonable in vivo estimate of final minimum lumen diameter. Mechanical performance studies were carried out in Korea Testing Laboratory (KTL). All experimental procedures followed the International Organization for Standardization (ISO) 25,539–2 or a standard test method (ASTM) F 2606–08. Compliance properties were measured outside the constrained region to reduce the effect of boundary condition [30].

### Animal study

The Ethics Committee of Chonnam National University Medical School and Chonnam National University Hospital approved the animal study for this research. Animal studies were performed with male New Zealand white rabbits (3.5 kg, obtained from Damool Science, Daejeon, Korea). The PCS was implanted to the rabbit iliac arteries. The stent was deployed by inflating the balloon and the resulting stent-to-artery ratio was 1.3:1. At every designated day of implantation, the vessels surrounding stents were isolated and fixed in 10% neutral buffered formalin. And then stents were subjected to fluorescence microscope analysis.

### Statistical analysis

Statistical analysis was performed with the aid of the commercially available software (SPSS Version 15, Chicago, IL, U.S.A.). The data were presented as mean value ± SD. Unpaired Student’s t test was used for the comparison of the two stent groups. A value of *p* < 0.05 was considered statistically significant.

## Results

### Surface morphologies of stent surface

**Fig. 1:**
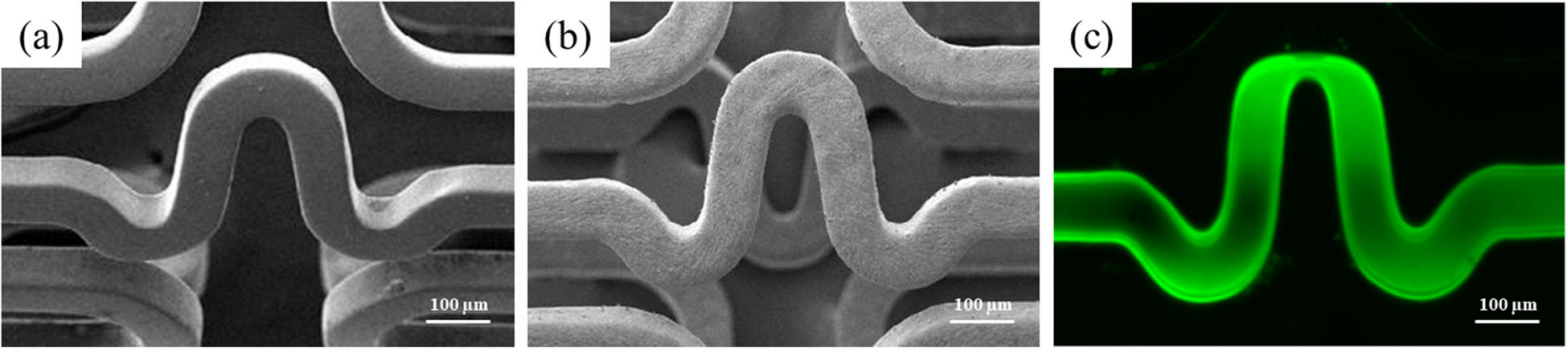
Surface morphologies of PCS. SEM images of before (a) and after (b) peptide coating procedure. A representative image taken under fluorescence microscopy showed FITC-conjugated PCS (c). All magnification of images was ser as 100 ×

The surface morphology that resulted from each coating step was investigated by SEM and fluorescence microscope. The surface morphology of PCS showed the well-coated and smoothen shape (Fig. 1b) compare to BMS (Fig. 1a). Significant FITC fluorescence was observed in strut of PCS (Fig. 1c).

### In vitro peptide disappearance

**Fig. 2:**
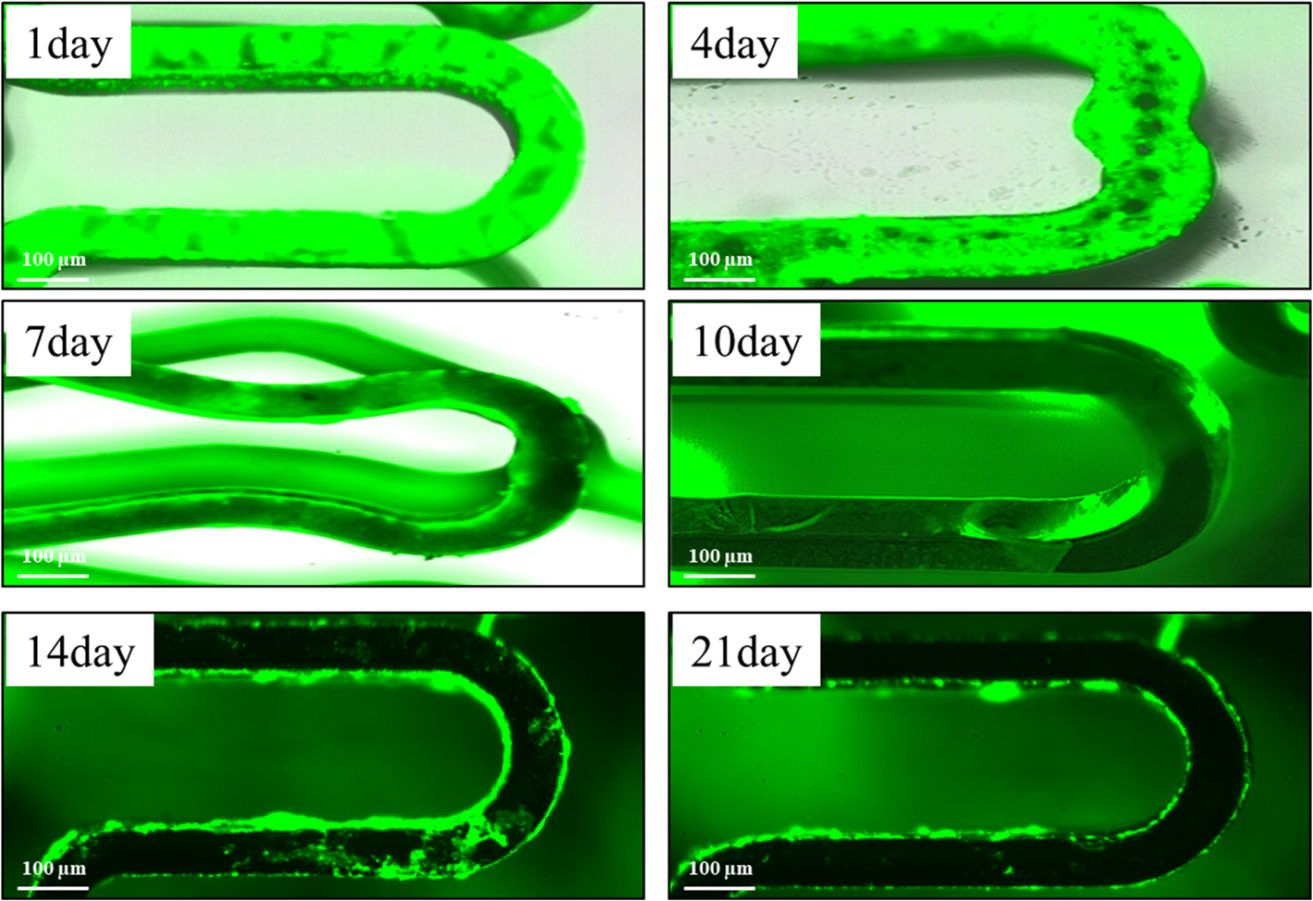
In vitro peptide disappearances on stent strut. The PCS was located at inside of silicon tube and PBS was circulated through silicon tube with 150 rpm. The PCS was taken out at every designated time point and subjected to fluorescence microscopy observation

The peptide was conjugated to dopamine which is the adhesive chemical. By the action of dopamine’s coordinate bond, peptide was coated to CNUH stent. Location of peptide was identified by FITC. Fluorescence was decreased and showed the blotched shape at 4 day of incubation. It was diminished gradually and was maintained to 21 day under the in vitro circulation system (Fig. 2).

### Effect of the peptide-coated stent on endothelial cell proliferation

**Fig. 3:**
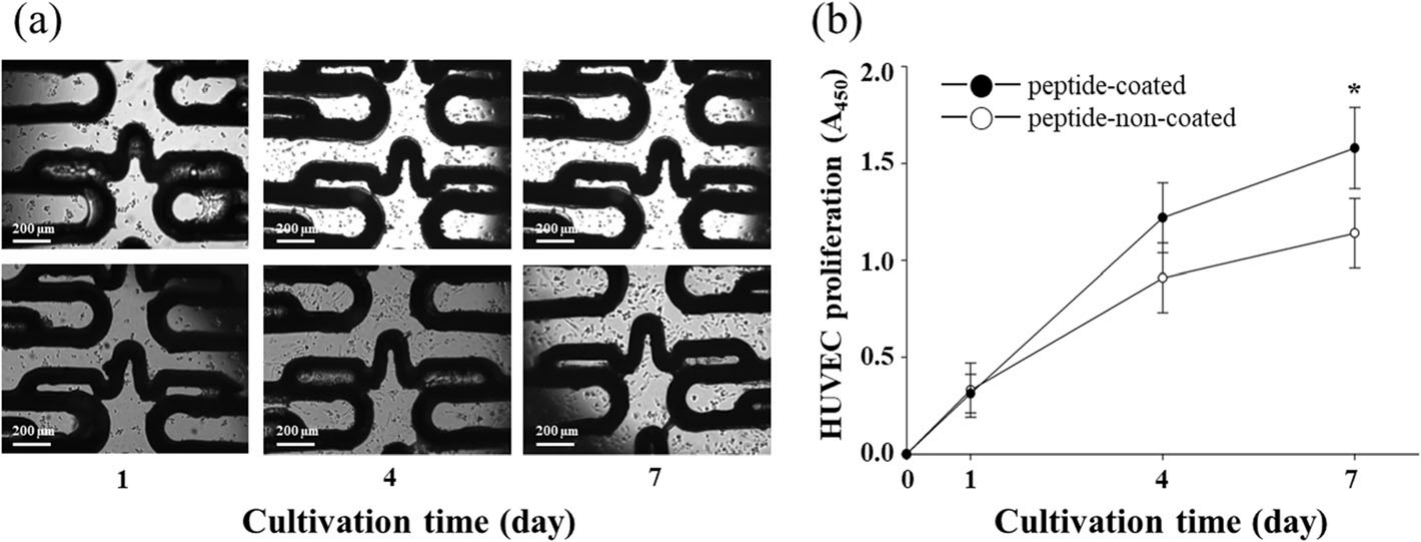
Proliferation of HUVEC. Cells were cultured in 24-well cell culture dish contained the 2-dimensional PCS and BMS. Results were obtained by XTT assay. Each datum point represents the mean ± SD (*n* = 10). *p < 0.05

Cell proliferation of HUVEC was investigated by XTT assay. Compare to BMS group, the proliferation of HUVEC on the PCS group was not influenced at 1 day of culture. The proliferation of HUVEC on the PCS group slightly improved at 4 day of culture (approximately 16.2 ± 3.52%). However, the proliferation of HUVEC was greatly increased in PCS group at 7 day of culture (approximately 36.4 ± 5.77%) (Fig. 3).

### Mechanical performance

#### Radial force

**Fig. 4:**
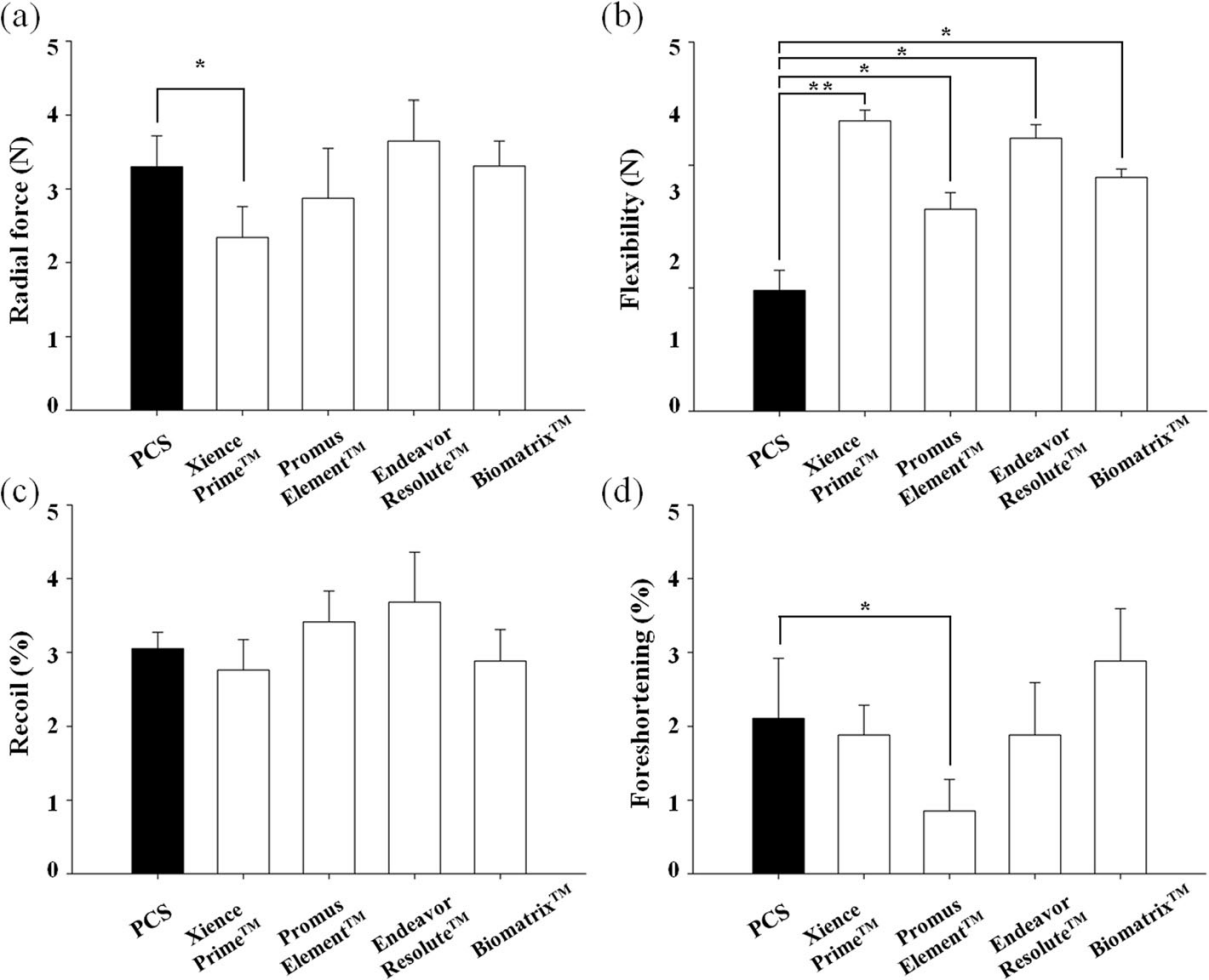
Mechanical properties of PCS and DESs. Radial force (a) and flexibility (b) were investigated by flat plate compression and 3-point bending test, respectively. Plastic deformation result of recoil (c) and foreshortening (d) was investigated under FDA guide line. Each datum point represents the mean ± SD (*n* = 10). *p < 0.05, **p < 0.005

In the flat plate radial compression test, The value of PCS was moderate (3.3 ± 0.42 N) compare to comparison DESs group (Xience Prime™; 2.3 ± 0.42 N, Promus Element™; 2.9 ± 0.68 N, Endeavor Resolute™; 3.7 ± 0.55 N and Biomatrix™; 3.3 ± 0.34 N) (Fig. 4a).

#### Flexibility - three point bending test

Flexibility result by 3-point bending test showed the greatest flexibility in PCS group (0.49 ± 0.082 N) among the competitors while the number of flexibility in others were lower than PCS group (Xience Prime™; 1.18 ± 0.044 N, Promus Element™; 0.82 ± 0.068 N, Endeavor Resolute™; 1.11 ± 0.055 N and Biomatrix™; 0.95 ± 0.034 N) (Fig. 4b).

#### Recoil and foreshortening

The value of recoil in PCS was moderate (3.1 ± 0.22%) compare to comparison DESs group (Xience Prime™; 2.8 ± 0.41 N, Promus Element™; 3.4 ± 0.42 N, Endeavor Resolute™; 3.7 ± 0.68 N and Biomatrix™; 2.9 ± 0.43 N) (Fig. 4c). The value of foreshortening in PCS showed middle level (2.1 ± 0.06%) in rage of competitor DESs (Xience Prime™; 1.9 ± 0.41 N, Promus Element™; 0.9 ± 0.43 N, Endeavor Resolute™; 1.9 ± 0.71 N and Biomatrix™; 2.9 ± 0.71 N) (Fig. 4d).

#### Compliance performance

The influence of the cell geometry upon the radial compliance of the stent is shown in Table 1. The nominal pressure (i.e. expansion to 3.0 mm in diameter) of PCS was 9 atm. It was similar to other DESs (Xience Prime™; 10 atm, Promus Element™; 12 atm, Endeavor Resolute™; 7 and Biomatrix™; 6).

**Table 1:** Compliance performance of various stents. Nominal pressure for each diameter indicated by bold font

Pressure		Stents				
Atm	kPa	PCS	Xience Prime™	Promus Element™	Endeavor Resolute™	Biomatrix™
6	608	2.73	2.67	2.56	2.95	3.00
7	709	2.86	2.77	2.63	3.00	3.03
8	811	2.97	2.87	2.72	3.05	3.06
9	912	3.02	2.95	2.81	3.10	3.09
10	1013	3.08	3.01	2.88	3.15	3.12
11	1115	3.15	3.08	2.95	3.20	3.15
12	1216	3.20	3.13	3.01	3.25	3.18
13	1317	3.25	3.19	3.06	3.30	3.21
14	1419	3.29	3.23	3.10	3.30	3.24

### In vivo peptide delivery

**Fig. 5:**
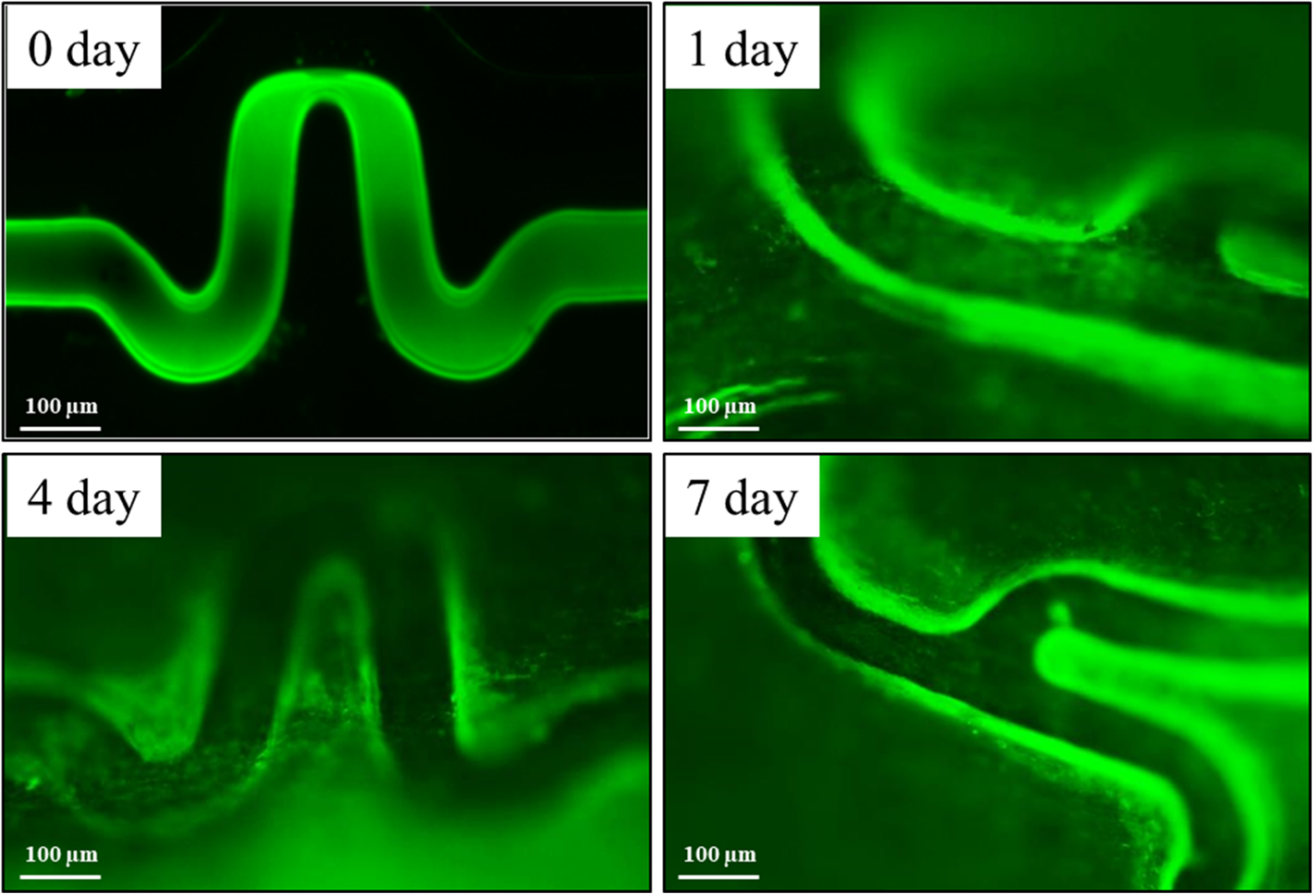
In vivo peptide delivery. The PCS was implanted to rabbit iliac arteries and the peptide delivery to vessels surrounding PCS was detected by FITC observation. At every designated time, the vessel surrounding PCS was isolated and subjected to fluorescence microscopy observation

The PCS was implanted to rabbit iliac arteries and the peptide delivery to vessels surrounding PCS was detected by FITC observation (Fig. 5). A green fluorescence layer was seen around stent strut. It was existing over the stent strut at early stage of implantation. However, the green color was decreased gradually and diminished completely at 7 day.

## Discussion

Previous investigations suggest that biomechanics has a key role in each of these pathobiologic stages. Moreover, the biomechanical impact of stents is largely governed by their exact design configuration. Selecting a stent design having appropriate mechanical properties to the vessels is an important parameter influencing the resulting wall coverage and strut positioning of several stenting techniques [31]. Clinical studies also have suggested restenosis has been correlated with geometric properties of stents, such as the number of struts, the strut width and thickness, and the geometry of the cross section of each strut [32–36]. These geometric properties play a key role in determining a stent’s overall mechanical properties, as well as the pressure loads that a stent can sustain when inserted into a native coronary artery. The successful deployment of stent is dependent on the clear understanding of its mechanical properties since the successful deployment of stent leads to a less damage on the targeting cardiovascular vessel, inducing a less restenosis which is known as one of the most serious problem occurred after percutaneous coronary intervention (PCI) [1, 37]. Since the force of stent after expansion is significantly related to the damage on the blood vessel that induces restenosis. In this study, specially synthesized peptide, WKYMVm, a selective FPR2 agonist for endothelial cell homing, was coated to our own designed CNUH stent. And its mechanical and physio-biological properties were investigated. As shown in Figs. 1, 2, and 5, location of peptide was identified by FITC. The peptide was conjugated to dopamine which is the adhesive chemical. By the action of dopamine’s coordinate bond, peptide was coated to CNUH stent. The peptide on stent was maintained to 21 day under in vitro circulation system (Fig. 2). Moreover, the peptide was delivered to vessel surrounding stent to 7 days in animal study (Fig. 5). Since, the endothelial healing process is continued from injured juncture by stent implantation, the maintaining and sustain delivery of peptide is reasonable. After introduction of the stents into the blood-contacting environment, the initial interaction between the stent surface and neighboring cells, especially endothelial cell, would affect the following cellular events on the surface. In addition, WKYMVm was reported as stimulating factor for ECFC [8]. Thus, the effect of the PCS on HUVEC proliferation was investigated. The result showed that the proliferation of HUVEC in PCS group was increased in PCS group at 7 day of culture compare to BMS (Fig. 3). This may occur because the inorganic material surface such as metal and even titanium provides an unfavorable environment for cell adhering and proliferation [38]. The in vitro result may be different from the results of in vivo experiments because it is difficult to define exact amount and kinds of enzymes or environment in body. Nevertheless, these results suggest not only non-cytotoxicity in vitro but also possibility of re-endothelialization in vivo. Higher radial force indicates better performance in practical situation. In the flat plate radial compression test, PCS showed moderate performance compare to commercialized DESs (Fig. 4a). It implicates the PCS is not inferior to other commercialized DES and PCS is able to apply to conventional balloon crimping. Especially, the flexibility of PCS (0.49 ± 0.082 N) was greatest among the competitor DESs (Fig. 4b). Previously, we have been reported the superiority of CNUH stent (0.46 ± 0.079 N) on flexibility [21, 22]. It was speculated that the mechanical properties of PCS is similar to CNUH since the peptide was coated to CNUH stent. Many DES essentially employ the same design configuration as their BMS counterparts with a drug-loaded polymer coating on the underlying metallic surface [20]. One of the major limitations of balloon angioplasty is early restenosis as a result of elastic recoil leading to vessel occlusion [39, 40]. The use of stents positioned in correspondence of the coronary vessel stenotic portion, emerged as an interesting therapeutic strategy due to the consistently reduction of restenosis occurrence with respect to the simple PCI. Although they can really prevent the coronary wall early elastic recoil and late re-modeling, two known events leading to restenosis, they induce in-stent restenosis due to the proliferation of vascular smooth muscle cell [41]. Our result showed that PCS did not show the outstanding performance in recoil and foreshortening test (3.1 ± 0.22% and 2.1 ± 0.06%, respectively) (Fig. 4c-d), which was the reasonable result under the guide line of FDA (less than 7.0%). The nominal pressure (3.0 mm in a diameter) of PCS established by compliance analysis was 9 atm (Table 1). The changing of PCS diameter by expansion was similar to other DESs, which is less than 10 atm of pressure for the nominal pressure. The difference in compliance suggests the significance of orientation of shape. The compliance of the expanded stent can be used to assess radial compliance-mismatch with the properties of the artery, found to be a contributing factor in acute injury to the artery and produce stress-induced growth and remodeling [42]. Compliance could be tailored better to match arteries by changing design parameters such as strut length, thickness, and unit cell geometry. The overall mechanical response of a stent can be suitably designed by changing the geometrical arrangement of struts in a unit cell.

## Conclusion

There is no doubt that PCS is enough competitive compared to the competitor on mechanical properties. Moreover, WKYMVm, specially synthesized peptide for stimulating endothelial cell proliferation, enhanced HUVEC proliferation. Since the PCS was fabricated as polymer–free process, secondary coating with polymer-based immunosuppressive drugs such as everolimus and biolimus may possible. Take together, PCS will be utilized for the fabricating the sequential eluting stent to inhibit restenosis and promote re-endothelialization in the future.

## Data Availability

Not applicable.

## Abbreviations

BMS: Bare metal stent
Co-Cr: Cobalt-choromium
DES: Drug-eluting stent
ECFC: Endothelial colongy-forming cell
HUVEC: Human umbilical vein endothelial cell
MRI: Magnetic resonance imaging
PCS: Peptide-coated stent

## References

[1] Serruys PW, Kutryk MJ, Ong AT (2006). Coronary-artery stents. N Engl J Med.

[2] Losordo DW, Isner JM, Diaz-Sandoval LJ (2003). Endothelial recovery: the next target in restenosis prevention. Circulation.

[3] Joner M, Finn AV, Farb A, Mont EK, Kolodgie FD, Ladich E, Kutys R, Skorija K, Gold HK, Virmani R (2006). Pathology of drug-eluting stents in humans: delayed healing and late thrombotic risk. J Am Coll Cardiol.

[4] Van Belle E, Tio FO, Chen D, Maillard L, Kearney M, Isner JM (1997). Passivation of metallic stents after arterial gene transfer of phVEGF165 inhibits thrombus formation and intimal thickening. J Am Coll Cardiol.

[5] Walter DH, Cejna M, Diaz-Sandoval L, Willis S, Kirkwood L, Stratford PW, Tietz AB, Kirchmair R, Silver M, Curry C, Wecker A, Yoon YS, Heidenreich R, Hanley A, Kearney M, Tio FO, Kuenzler P, Isner JM, Losordo DW (2004). Local gene transfer of phVEGF-2 plasmid by gene-eluting stents: an alternative strategy for inhibition of restenosis. Circulation.

[6] Le Y, Gong W, Li B, Dunlop NM, Shen W, Su SB, Ye RD, Wang JM (1999). Utilization of two seven-transmembrane, G protein-coupled receptors, formyl peptide receptor-like 1 and formyl peptide receptor, by the synthetic hexapeptide WKYMVm for human phagocyte activation. J Immunol.

[7] Seo JK, Bae YS, Song H, Baek SH, Kim BS, Choi WS, Suh PG, Ryu SH (1998). Distribution of the receptor for a novel peptide stimulating phosphoinositide hydrolysis in human leukocytes. Clin Biochem.

[8] Heo SC, Kwon YW, Jang IH, Jeong GO, Yoon JW, Kim CD, Kwon SM, Bae YS, Kim JH (2014). WKYMVm-induced activation of formyl peptide receptor 2 stimulates ischemic neovasculogenesis by promoting homing of endothelial colony-forming cells. Stem Cells.

[9] Miao Z, Premack BA, Wei Z, Wang Y, Gerard C, Showell H, Howard M, Schall TJ, Berahovich R (2007). Proinflammatory proteases liberate a discrete high-affinity functional FPRL1 (CCR12) ligand from CCL23. J Immunol.

[10] Halwani DO, Anderson PG, Brott BC, Anayiotos AS, Lemons JE (2012). The role of vascular calcification in inducing fatigue and fracture of coronary stents. J Biomed Mater Res B Appl Biomater.

[11] Nakazawa G, Finn AV, Vorpahl M, Ladich E, Kutys R, Balazs I, Kolodgie FD, Virmani R (2009). Incidence and predictors of drug-eluting stent fracture in human coronary artery a pathologic analysis. J Am Coll Cardiol.

[12] Hiltrop N, De Cock D, Ferdinande B, Adriaenssens T (2014). Detailed in vivo visualization of stent fracture causing focal restenosis using 3D reconstruction software for high-resolution optical coherence tomography images. Eur Heart J Cardiovasc Imaging.

[13] Kastrati A, Schomig A, Dirschinger J, Mehilli J, von Welser N, Pache J, Schuhlen H, Schilling T, Schmitt C, Neumann FJ (2000). Increased risk of restenosis after placement of gold-coated stents: results of a randomized trial comparing gold-coated with uncoated steel stents in patients with coronary artery disease. Circulation.

[14] Rogers C, Tseng DY, Squire JC, Edelman ER (1999). Balloon-artery interactions during stent placement: a finite element analysis approach to pressure, compliance, and stent design as contributors to vascular injury. Circ Res.

[15] Dyet JF, Watts WG, Ettles DF, Nicholson AA (2000). Mechanical properties of metallic stents: how do these properties influence the choice of stent for specific lesions?. Cardiovasc Intervent Radiol.

[16] Hanawa T (2009). Materials for metallic stents. J Artif Organs.

[17] Lewis G (2008). Materials, fluid dynamics, and solid mechanics aspects of coronary artery stents: a state-of-the-art review. J Biomed Mater Res B Appl Biomater.

[18] Mani G, Feldman MD, Patel D, Agrawal CM (2007). Coronary stents: a materials perspective. Biomaterials.

[19] Muller-Hulsbeck S, Schafer PJ, Charalambous N, Yagi H, Heller M, Jahnke T (2010). Comparison of second-generation stents for application in the superficial femoral artery: an in vitro evaluation focusing on stent design. J Endovasc Ther.

[20] Lemos PA, Moulin B, Perin MA, Oliveira LA, Arruda JA, Lima VC, Lima AA, Caramori PR, Medeiros CR, Barbosa MR, Brito FS, Ribeiro EE, Martinez EE (2009). Randomized evaluation of two drug-eluting stents with identical metallic platform and biodegradable polymer but different agents (paclitaxel or sirolimus) compared against bare stents: 1-year results of the PAINT trial. Catheter Cardiovasc Interv.

[21] Lim KS, Bae IH, Kim JH, Park DS, Kim JM, Sim DS, Hong YJ, Jeong MH (2013). Mechanical and Histopathological comparison between commercialized and newly designed coronary bare metal stents in a porcine coronary restenosis model. Chonnam Med J.

[22] Bae IH, Lim KS, Park JK, Park DS, Lee SY, Jang EJ, Ji MS. Mechanical behavior and in vivo properties of newly designed bare metal stent for enhanced flexibility. J Indust Eng Chem in press. 2014.

[23] Buszman P, Trznadel S, Zurakowski A, Milewski K, Kinasz L, Krol M, Kondys M (2007). Prospective registry evaluating safety and efficacy of cobalt-chromium stent implantation in patients with de novo coronary lesions. Kardiol Pol.

[24] Seo JK, Choi SY, Kim Y, Baek SH, Kim KT, Chae CB, Lambeth JD, Suh PG, Ryu SH (1997). A peptide with unique receptor specificity: stimulation of phosphoinositide hydrolysis and induction of superoxide generation in human neutrophils. J Immunol.

[25] You I, Kang SM, Byun Y, Lee H (2011). Enhancement of blood compatibility of poly (urethane) substrates by mussel-inspired adhesive heparin coating. Bioconjug Chem.

[26] Bae IH, Park IK, Park DS, Lee H, Jeong MH (2012). Thromboresistant and endothelialization effects of dopamine-mediated heparin coating on a stent material surface. J Mater Sci Mater Med.

[27] Gilles MA, Hudson AQ, Borders CL (1990). Stability of water-soluble carbodiimides in aqueous solution. Anal Biochem.

[28] Ma X, Oyamada S, Gao F, Wu T, Robich MP, Wu H, Wang X, Buchholz B, McCarthy S, Gu Z, Bianchi CF, Sellke FW, Laham R (2011). Paclitaxel/sirolimus combination coated drug-eluting stent: in vitro and in vivo drug release studies. J Pharm Biomed Anal.

[29] Twentyman PR, Luscombe M (1987). A study of some variables in a tetrazolium dye (MTT) based assay for cell growth and chemosensitivity. Br J Cancer.

[30] Berry JL, Manoach E, Mekkaoui C, Rolland PH, Moore JE, Rachev A (2002). Hemodynamics and wall mechanics of a compliance matching stent: in vitro and in vivo analysis. J Vasc Interv Radiol.

[31] Mortier P, Van Loo D, De Beule M, Segers P, Taeymans Y, Verdonck P, Verhegghe B (2008). Comparison of drug-eluting stent cell size using micro-CT: important data for bifurcation stent selection. EuroIntervention.

[32] Kastrati A, Mehilli J, Dirschinger J, Dotzer F, Schuhlen H, Neumann FJ, Fleckenstein M, Pfafferott C, Seyfarth M, Schomig A (2001). Intracoronary stenting and angiographic results: strut thickness effect on restenosis outcome (ISAR-STEREO) trial. Circulation.

[33] Garasic JM, Edelman ER, Squire JC, Seifert P, Williams MS, Rogers C (2000). Stent and artery geometry determine intimal thickening independent of arterial injury. Circulation.

[34] Lau KW, Johan A, Sigwart U, Hung JS (2004). A stent is not just a stent: stent construction and design do matter in its clinical performance. Singap Med J.

[35] McLean DR, Eiger NL (2002). Stent design: implications for restenosis. Rev Cardiovasc Med.

[36] Morton AC, Crossman D, Gunn J (2004). The influence of physical stent parameters upon restenosis. Pathol Biol (Paris).

[37] Kastrati A, Mehilli J, Dirschinger J, Pache J, Ulm K, Schuhlen H, Seyfarth M, Schmitt C, Blasini R, Neumann FJ, Schomig A (2001). Restenosis after coronary placement of various stent types. Am J Cardiol.

[38] Popat KC, Leoni L, Grimes CA, Desai TA (2007). Influence of engineered titania nanotubular surfaces on bone cells. Biomaterials.

[39] Tesfamariam B (2007). Local vascular toxicokinetics of stent-based drug delivery. Toxicol Lett.

[40] Tanimoto S, Bruining N, van Domburg RT, Rotger D, Radeva P, Ligthart JM, Serruys PW (2008). Late stent recoil of the bioabsorbable everolimus-eluting coronary stent and its relationship with plaque morphology. J Am Coll Cardiol.

[41] Meng S, Liu Z, Shen L, Guo Z, Chou LL, Zhong W, Du Q, Ge J (2009). The effect of a layer-by-layer chitosan-heparin coating on the endothelialization and coagulation properties of a coronary stent system. Biomaterials.

[42] Moore J, Berry JL (2002). Fluid and solid mechanical implications of vascular stenting. Ann Biomed Eng.

